# The Protective Effect of Ellagic Acid and Its Metabolites Against Organ Injuries: A Mitochondrial Perspective

**DOI:** 10.1002/fsn3.70077

**Published:** 2025-04-09

**Authors:** Sohrab Rahmani, Ali Roohbakhsh, Yazdan Hasani Nourian, Gholamreza Karimi

**Affiliations:** ^1^ Chemical Injuries Research Center, Systems Biology and Poisonings Institute Baqiyatallah University of Medical Sciences Tehran Iran; ^2^ Student Research Committee Mashhad University of Medical Sciences Mashhad Iran; ^3^ Department of Pharmacodynamics and Toxicology, School of Pharmacy Mashhad University of Medical Sciences Mashhad Iran; ^4^ Institute of Pharmaceutical Technology, Pharmaceutical Research Center Mashhad University of Medical Sciences Mashhad Iran

**Keywords:** apoptosis, ellagic acid, mitochondria, oxidative stress

## Abstract

Mitochondria are essential for maintaining health, and dysfunction of them leads to various diseases. Their role is not limited to energy production but serves multiple mechanisms varying from calcium hemostasis, reactive oxygen species production, and regulation of apoptotic cell death. In recent years, several strategies have been developed to preserve mitochondria. Ellagic acid (EA) is a polyphenol extracted from many plants. The intestinal microflora converts EA to urolithins with high bioavailability. EA and urolithins exhibit mitochondrial‐protective effects by regulating mitochondrial complexes, sirtuins, mitophagy, and mitochondrial antioxidant enzymes. This review highlights the mito‐protective effects of EA and urolithins on mitochondrial injuries induced by various drugs and toxic compounds.

## Graphical abstract

Ellagic acid (EA) is a natural compound and its gut microbiota derivatives (urolithins) play a crucial role in the treatment of multiple diseases. This review highlights the mito‐protective effects of EA and urolithins on mitochondrial injuries induced by various drugs and toxic compounds.

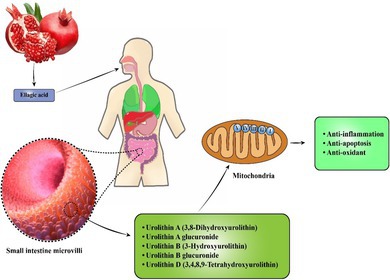

**Abbreviations**
AD: Alzheimer's disease
ALT: Alanine aminotransferase
ARE: Antioxidant Response Element
AST: Aspartate aminotransferase
ATP: Adenosine triphosphate
Bax: Bcl‐2‐associated X protein
Bcl‐2: B‐cell lymphoma 2
BNIP3: Bcl‐2 interacting protein 3
CAT: Catalase
CKD: Chronic kidney disease
CVD: Cardiovascular diseases
Dox: Doxorubicin
Drp‐1: Dynamin‐related protein 1
EA: Ellagic acid
ERR: Estrogen‐related receptor
FOXO1: Forkhead box transcription factor1
GPx: Glutathione peroxidase
GRx: Glutathione reductase
GSH: Glutathione
GST: Glutathione‐S‐transferase
HO‐1: Hemoxygenase‐1
ICDH: Isocitrate dehydrogenase
IL‐6: Interleukin‐6
ISO: Isoproterenol
Keap1: Kelch‐like ECH‐associated protein1
MDA: Malondialdehyde
MDH: Malate dehydrogenase
MDH: Malate dehydrogenase
MMP: Mitochondrial membrane potential
mPTP: mitochondrial permeability transition pore
MS: Multiple sclerosis
mtROS: mitochondrial reactive oxygen species
NF‐κB: Nuclear factor‐kappaB
Nrf2: Nuclear factor erythroid 2‐related factor 2
Nrf2: Nuclear factor erythroid 2‐related factor 2
PD: Parkinson's disease
PGC‐1α: Peroxisome proliferator‐activated receptor gamma
SDH: Succinate dehydrogenase
SOD: Superoxide dismutase
STING: Stimulator of interferon gene
UA: Urolithin A
α‐KGDH: α‐ketoglutarate dehydrogenase

## 1 Introduction

Mitochondria are essential double‐membrane organelles in the cytoplasm that play critical physiological roles in human cells, including cellular energy production, redox metabolism, calcium homeostasis, and regulation of apoptosis(Garcia‐Souza and Oliveira 2014; Sohrab Rahmani and Rezaei 2020). Recent studies indicate that mitochondria play a significant role in various diseases, including cancer, metabolic disorders, aging, and neurodegenerative diseases such as Alzheimer's (AD) and Parkinson's diseases (PD). The electron transport chain of mitochondria is a crucial source for generating free radicals (Rahmani et al. 2023b, 2024a). Any disturbance in mitochondrial function can trigger a rise in the production of free radicals in the body (Rahmani et al. 2023b, 2024a). This, in turn, can lead to diseases. The primary roles of mitochondria in cells are adenosine triphosphate (ATP) generation, fatty acid metabolism, and steroid synthesis. Moreover, these organelles regulate calcium signaling and apoptosis (Rahmani et al. 2023b). In recent decades, many studies have consistently highlighted mitochondria as the critical targets of notorious toxic substances such as arsenic (Firdaus et al. 2018a, 2018b), acrolein (Shafie et al. 2021), and chromium (Cuevas‐Magaña et al. 2022). These findings highlight the critical role of mitochondria in toxicological responses and the potentially harmful effects of chemical substances on this organelle. Therefore, it is crucial to conduct mechanistic studies to understand the effects of chemical substances on mitochondria and develop strategies to protect these essential organelles. Accordingly, recent studies have provided compelling evidence for the biological activity of natural compounds. These natural compounds are promising for treating various diseases, including neurological complications, cancers, metabolic diseases, and cardiovascular ailments (Çömez et al. 2020; Helli et al. 2024; Rahmani et al. 2023a; Wang et al. 2022b). Notably, researchers are now exploring the significant role of natural compounds in regulating mitochondrial functions (Rahmani et al. 2023b, 2024b).

**FIGURE 1:**
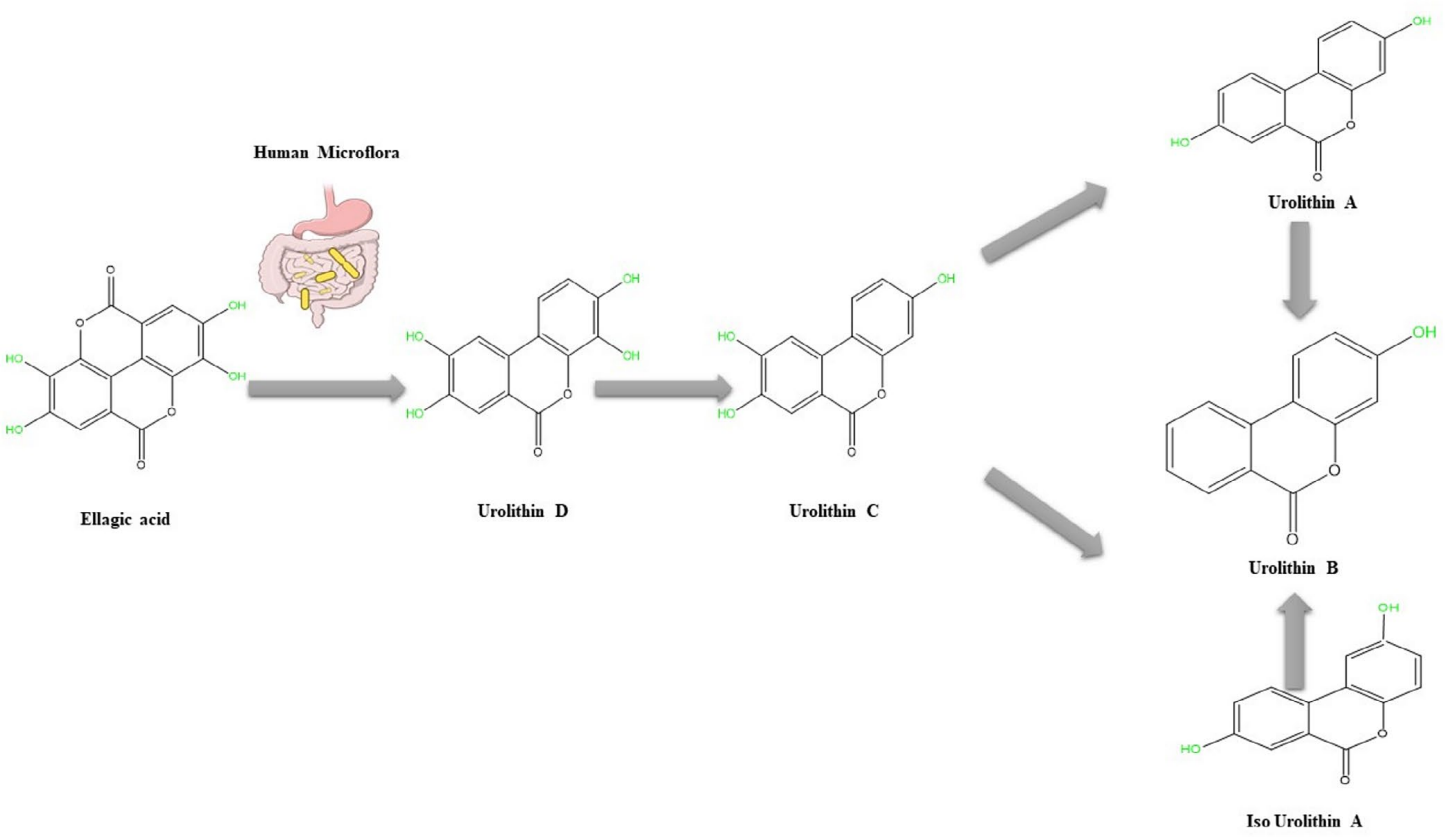
A schematic view of ellagic acid and its gut microbiota metabolism pathway.

Ellagic acid (EA) is a naturally occurring polyphenol found in fruits such as pomegranates, grapes, and strawberries. It has been extensively studied for its pharmacological effects, including anti‐inflammatory, antiviral, antioxidant, and anxiolytic properties (Jamshidi et al. 2023). EA is composed of lactone and hydroxyl groups, essential for its antioxidant abilities (Figure 1). However, its hydrophobic nature limits its bioavailability in animals and humans. On the other hand, the bioavailability of EA's metabolites, known as urolithins, is greater than that of the original compound. Interestingly, both EA and urolithins exhibit significant antioxidant activity. Numerous studies have demonstrated that EA targets mitochondria, resulting in mitochondrial‐protective effects in experimental models. This review article provides an overview of the protective effects of EA and its metabolite, urolithin, on mitochondria, focusing on their role in minimizing tissue damage by preventing dysregulation of this micro‐organelle (Tables 1 and 2).

**TABLE 1:** Summary of the mitochondrial‐protective effects of ellagic acid and urolithins in in vitro studies.

In vitro model	Ellagic acid/urolithins (dose, route of exposure, duration)	Toxic agent (dose, route of exposure, duration)	Findings	Reference
Isolated rat liver mitochondria	EA, 20, 40, and 80 μM, for 1 h	As2O3, 20, 40, and 100 μM, for 1 h	Decreased ROS, MDA, MMP Increased complex II activity, GSH content	(Keshtzar et al. 2016)
Isolated rat heart mitochondria	EA, 10–100 μM, for 60 min	Bevacizumab, 50 and 100 mg/mL, for 60 min	Decreased mitochondrial swelling, ROS, and MMP Increased complex II activity	(Khanlou et al. 2022)
SH‐SY5Y cells	EA,10 and 20 M, for 60 min	As2O3, 2 M	Decreased ROS, LDH, MMP, and cytochrome c	(Firdaus et al. 2018b)
Isolated human lymphocytes	EA, 10, 25, and 50 M, for 4 h	Acrylamide, 50 M, for 4 h	Decreased ROS, MDA, and MMP Increased GSH content	(Salimi et al. 2021)
Isolated adult rat ventricular cardiomyocytes	EA, 10, 20, 50 μM, for 4 h	Clozapine, 50 μM, for 4 h	Decreased ROS, MDA, MMP and lysosomal damages Increased GSH content	(Ahangari et al. 2022)
Isolated cardiomyocytes mitochondria	EA, 10, 50, and 100 μM	Celecoxib, 16 μg/mL	Decreased ROS, MDA, MMP, SDH, and mitochondrial swelling Increased GSH content	(Atashbar et al. 2021)

**TABLE 2:** Summary of the mitochondrial‐protective effects of ellagic acid and urolithins in in vivo studies.

In vivo model	EA (dose, route of exposure, duration)	Toxic agent (dose, route of exposure, duration)	Findings	Reference
Methotrexate hepatotoxicity, Wistar rats	EA, 5 mg/kg, oral, for 10 days	Methotrexate, 20 mg/kg, i.p., single dose	Decreased MDA, ROS, MMP collapse, cytochrome c, caspases‐3/9, IL‐6, and NF‐ĸB, Increased SOD, GSH, Nrf2, and HO‐1	(Ebrahimi et al. 2019)
Doxorubicin hepatotoxicity, Wistar rats	UA, 2.5 and 5 mg/kg, i.p., for 7 days	Doxorubicin, 20 mg/kg, i.p., single dose	Decreased cytochrome c and caspase‐3	(Shahid Karim et al. 2023)
Chromium nephrotoxicity, Wistar rats	EA, 15 and 30 mg/kg, oral, for 10 days	Chromium, 15 mg/kg, s.c., single dose	Decreased MDA, ROS, MMP, and TNF‐α	(Cuevas‐Magaña et al. 2022)
Gentamicin nephrotoxicity, Sprague Dawley rats	EA, 10 mg/kg, oral, for 10 days	Gentamicin, 100 mg/kg, i.p., for 10 days	Decreased MDA, ROS, MMP loss, cytochrome c, and Bax Increased SOD, GSH, CAT, and Bcl‐2	(Sepand et al. 2016)
Ifosfamide nephrotoxicity, Wistar rats	EA, 10 mg/kg, i.p., for 2 days	Ifosfamide, 500 mg/kg, i.p., single dose	Decreased MDA, ROS, and MMP loss Increased SDH, SOD, and GSH	(Shabani et al. 2023)
Isoproterenol cardiotoxicity, Wistar rats	EA, 7.5,15 mg/kg, oral, for 10 days	Isoproterenol, 100 mg/kg, s.c., twice at an interval of 24 h	Decreased MDA, MMP loss Increased GPx, GR, GST, ICDH, SDH, MDH, α‐KGDH	(Kannan and Quine 2012)
Cuprizone neurotoxicity, C57BL/6 mice	EA, 0.2% w/w, Oral, for 42 days	Cuprizone, 5, 50, and 100 mg/kg, Oral, for 42 days	Decreased MDA, ROS, and MMP loss Increased complex II, III, IV, and SI	(Khodaei et al. 2019)
Arsenic neurotoxicity, Wistar rats	EA, 20 and 40 mg/kg, Oral, for 11 days	Arsenic, 10 mg/kg, Oral, for 8 days	Decreased ROS, MMP loss IL‐1β, TNF‐α, INFγ	(Firdaus et al. 2018a)
Rotenone neurotoxicity, *Drosophila melanogaster*	EA, 3 and 5 mg/kg, Oral, for 7 days	Rotenone, 500 μM, Oral, for 7 days	Decreased MDA, HO, and NO Increased complex I, II, activity, CAT, and GSH	(Adedara et al. 2023)
Alzheimer's disease model, APP/PS1 mice	UA, 200 mg/kg/day, Oral, for 5 months	—	Increased Parkin Decreased Bax and PINK1	(Hou et al. 2024)
Chronic kidney disease model, C57BL/6 mice	UA, 50 and 100 mg/kg, Oral, for 8 weeks	—	Increased PINK1, Parkin, LC3, and p62 Decreased STING, NLRP3, caspase‐1 p20, and IL‐1β	(Zhang et al. 2022)
Metabolic cardiomyopathy model, C57BL/6 mice	UA, 50 mg/kg, Oral, for 4 weeks	—	Increased Pink1, Park2, MFN1, Atg9, and Rab7	(Huang et al. 2023)
Diabetic cardiomyopathy model, Wistar rats	UA, 2.5 mg/kg, i.p., for 8 weeks	Streptozotocin, 65 mg/kg, i.p.	Increased Nrf2, SIRT1, GSH, MnSOD Decreased ROS, Bax, cleaved caspaspe3, TNF‐α	(Albasher et al. 2022)
Ischemic stroke model, C57BL/6 mice	UA, 1.5 and 2 mg/kg	—	Increased Bcl‐2 Decreased Bax and caspase‐3	(Lin et al. 2020)

### 1.1 The Effect of EA on Important Protein Signaling Pathways

Recent studies shed light on the impact of EA on different protein signaling pathways. In antioxidant‐related pathways, regardless of its scavenging activity due to the presence of four hydroxyl and two lactone groups, it has been shown that EA could enhance the Keap1/Nrf2/ARE signaling pathway (Naghibi et al. 2023; Wang et al. 2022a; Zhu et al. 2022). Nrf2 is a redox‐sensitive transcription factor that serves as the primary regulator of the antioxidant response within the cell. Under conditions of stress or toxicity, Nrf2 moves into the nucleus, where it binds to DNA and initiates the transcription of genes associated with the antioxidant defense system (Ríos et al. 2018). Moreover, recent studies show that EA is able to possess antioxidant effects by regulation of sirtuins, specifically sirtuin 1 (SIRT1) and sirtuin 3 (SIRT3) (Naghibi et al. 2023; Wang et al. 2024). SIRT1 is a histone deacetylase protein that is able to sense energy levels in cells. It can help cells to resist external stress and improve metabolism. Recent studies show that SIRT1 could activate Nrf2 through deacetylation leading to regulation of its downstream antioxidant and detoxification gene. Various examinations have established that EA exhibits notable anti‐inflammatory properties (Wang et al. 2024). studies suggest that EA may exert its anti‐inflammatory effects by inhibiting pro‐inflammatory cytokines, including interferon‐γ (IFN‐γ), interleukin‐1 (IL‐1), interleukin‐6 (IL‐6), and interleukin‐17 (IL‐17). Furthermore, EA has been shown to effectively block the nuclear factor kappa‐light‐chain‐enhancer of activated B cells (NF‐κB) pathway, a pivotal mechanism in the inflammatory response. In addition, multiple studies have indicated that EA may alleviate apoptosis by modulating the expression of proteins such as Bcl‐2–associated X protein (Bax), B‐cell lymphoma 2 (Bcl‐2), and caspase 3 and 9 (Wang et al. 2024).

### 1.2 The Anticancer Activity of EA

Cancer is a complex and multifactorial disease that poses a significant challenge to public health worldwide, exhibiting a high incidence and mortality rate across diverse populations. Despite advancements in cancer treatment, more effective drugs with fewer side effects are still urgently needed (Pourbarkhordar et al. 2024). Recent research indicates that EA has the potential to inhibit cell proliferation and may play a role in counteracting the development of cancer. In light of this perspective, some studies also showed that EA specifically could ameliorate cancer development through mitochondrial‐related pathway interactions. It has been shown that EA treatment in a model of lung cancer was able to inhibit cell proliferation and reduce ATP levels (Duan et al. 2020). Another study showed that EA induced cell cycle arrest and reduced cell proliferation in a model of colorectal and breast cancers. It was indicated that the mechanism underlying the anticancer effects of EA was related to the inhibition of the mitochondrial dynamic protein (Drp‐1) (Yakobov et al. 2023).

### 1.3 Mito‐Protective Effect of EA on the Hepato‐Renal System

#### 1.3.1 Acrolein Hepatotoxicity

Acrolein is a harmful unsaturated aldehyde widely found in the environment. Humans can be exposed to acrolein through various sources such as food, water, and even smoking cigarettes (Mohammad et al. 2012). It has been suggested that acrolein could play a role in hepato‐renal and neuronal toxicities. Acrolein affects several cell components, such as DNA, proteins, and mitochondria (Mohammad et al. 2012). Mitochondrial disruption has been reported in previous studies. It has been reported that acrolein in high doses interferes with mitochondrial function by dysregulating mitochondrial complexes and mitochondrial‐related enzymes such as pyruvate dehydrogenase and α‐ketoglutarate dehydrogenase (Moghe et al. 2015; Shafie et al. 2021). An in vitro study on isolated rat livers of mitochondria revealed that EA treatment reduced mitochondrial damage induced by acrolein, indicating that ROS and MMP levels were reduced. However, no significant effects on ATP, GSH, or complex II function were observed (Shafie et al. 2021). The authors claimed that antioxidant properties did not protect against all mitochondrial toxicants (Shafie et al. 2021).

#### 1.3.2 Arsenic Hepatotoxicity

Arsenic is a highly toxic metalloid that causes acute and chronic hepatotoxicity in both organic and inorganic forms. Long‐term arsenic exposure has been associated with various diseases, including lung and skin cancers, diabetes, cognitive impairment, and hepatomegaly (Keshtzar et al. 2016). Arsenic's toxicity mechanism is not fully understood. However, previous studies have highlighted the role of mitochondria in arsenic toxicity. Arsenate (As^5+^), due to its structural similarity to phosphate, can interfere with ATP synthesis by substituting for phosphate. Since mitochondria are the main source of ATP production in cells, a previous study by Keshtzar et al. on isolated rat liver mitochondria demonstrated that arsenic exposure disrupted mitochondrial ATP production. Moreover, they reported that arsenic exposure could inhibit complexes I and II, resulting in ROS overproduction. EA treatment dose‐dependently reduced ROS and MDA levels. Indeed, EA pretreatment reduced mitochondrial membrane damage (Keshtzar et al. 2016).

#### 1.3.3 Methotrexate Hepatotoxicity

Methotrexate is a drug used for treating malignancies, rheumatoid arthritis, leukemia, and psoriasis (Cronstein 1997). However, it may also affect the proliferation of normal cells, such as hepatic cells. Methotrexate hepatotoxicity restricts its long‐term clinical use. The exact mechanism(s) of methotrexate hepatotoxicity remain unclear (Bath et al. 2014). The oxidative stress caused by methotrexate is believed to damage mitochondria. Since mitochondria are the main source of ROS, damaged mitochondria could produce excessive ROS. Increased ROS levels trigger the formation of mitochondrial permeability transition pores (mPTP), which disrupt mitochondrial membrane potential (MMP) and initiate the early apoptotic pathway by releasing cytochrome c into the cytoplasm. In this regard, Ebrahimi et al. demonstrated that rats treated with methotrexate developed hepatotoxicity, which was attributed to ROS overproduction. Moreover, methotrexate treatment altered the levels of enzymatic and non‐enzymatic antioxidants like superoxide dismutase (SOD) and glutathione (GSH). Seemingly, these effects were either attributed to the direct antioxidant properties of EA or indirect effects on the Nrf2/HO‐1 signaling pathway, which reduced ROS overproduction. The researchers evaluated mitochondrial markers like mitochondrial swelling, MMP, and cytochrome c levels. Interestingly, all the above‐mentioned markers were improved by EA treatment. Additionally, EA blocked the apoptotic pathway by downregulation of Bax and upregulation of Bcl‐2 proteins (Ebrahimi et al. 2019).

#### 1.3.4 Chromium Nephrotoxicity

Chromium is a highly toxic heavy metal found in two main forms: trivalent chromium (III) and hexavalent chromium (VI) (Hegazy et al. 2016). Hexavalent chromium is a chemical compound widely used in various industries, including stainless steel manufacturing, chrome plating, leather tanning, welding, and wood processing. However, exposure to hexavalent chromium‐containing compounds, whether in the workplace or the environment, can be incredibly harmful and carcinogenic to both humans and animals. Because chromium is mainly excreted through the kidneys (Hegazy et al. 2016), nephrons are susceptible to chromium toxicity. Chromium's toxic effects are mainly due to oxidative stress, causing severe damage to vital organs (Hegazy et al. 2016). A recent study indicated that EA treatment could preserve mitochondria from chromium damage. It also improved mitochondrial enzyme function and mitochondrial respiration (Cuevas‐Magaña et al. 2022).

#### 1.3.5 Gentamicin Nephrotoxicity

Gentamicin is a widely used antibiotic for Gram‐negative bacterial infections. However, its nephrotoxicity limits its clinical use (Katary and Salahuddin 2017). Several studies highlight the role of mitochondria in gentamicin‐induced nephrotoxicity. An in vivo study found that EA treatment could prevent oxidative stress by activating antioxidant enzymes such as CAT, SOD, and GSH. Furthermore, EA reduced apoptosis through Bcl‐2 upregulation and Bax downregulation. Additionally, EA reduced mitochondrial ROS content, mitochondrial swelling, cytochrome c release, and MMP loss (Sepand et al. 2016).

#### 1.3.6 Ifosfamide Nephrotoxicity

Ifosfamide is an effective anticancer drug used for various cancer types. Numerous studies have linked ifosfamide use to nephrotoxicity (Quiroz‐Aldave et al. 2024). It seems that the metabolite of ifosfamide is the main culprit for nephrotoxicity. A recent study suggests that EA could reduce ifosfamide nephrotoxicity by regulating mitochondrial parameters. EA treatment enhanced mitochondrial succinate dehydrogenase activity (SDH) and MMP. Moreover, EA reduced mitochondrial ROS formation (Shabani et al. 2023).

### 1.4 Mito‐Protective Effect of Urolithins on the Hepato‐Renal System

#### 1.4.1 Acetaminophen Hepatotoxicity

Acute liver injury is a prevalent clinical condition attributed to a range of pathological factors, most notably oxidative stress and inflammation. These factors play a significant role in the progression of liver damage. The condition is characterized by liver cell necrosis and a failure to regenerate liver cells. In severe cases, this can progress to liver failure and hemorrhage, which pose a serious threat to the health and safety of patients(Li et al. 2025). Acetaminophen overdose is the most frequent cause of acute liver failure in the USA and many other countries (Li et al. 2022). Acetaminophen is an effective and easily available antipyretic analgesic drug. While acetaminophen is a safe and effective drug at recommended doses, it may cause hepatotoxicity and acute liver failure with overdose (Lancaster et al. 2015). Mitochondria are one of the main targets of acetaminophen toxicity as it disrupts ATP production, inhibits mitochondrial complex II, and increases cytochrome c release (Lancaster et al. 2015). It has been shown that urolithin A (UA), by induction of the mitochondrial autophagy process (mitophagy) and Nrf2/ARE signaling, protects the liver against acetaminophen toxicity (Gao et al. 2022).

#### 1.4.2 Doxorubicin Hepatotoxicity

As an effective anticancer agent, doxorubicin (Dox) usually harms cells that are not malignant. Hepatic injury caused by Dox has been reported in patients during chemotherapy (Karim et al. 2023). Oxidative stress, mitochondrial dysfunction, and apoptotic factors are considered the main reasons for Dox‐induced hepatotoxicity (Karim et al. 2023). A recent study showed that UA treatment protected the liver by suppressing apoptosis and regulating the mitochondrial apoptotic pathway. Moreover, UA treatment reduced caspase‐3 and cytochrome c oxidase levels (Karim et al. 2023).

#### 1.4.3 Chronic Kidney Disease

Chronic kidney disease (CKD) is a global public health issue defined as persistent alterations in kidney structure and function with adverse outcomes like end‐stage renal and cardiovascular diseases (Jankowski et al. 2021). Renal injury and metabolic stress in CKD are closely linked to mitochondrial dysfunction (Mafra et al. 2018; Zeng et al. 2023). It has been documented that mitochondrial damage‐induced mitochondrial DNA (mtDNA) leakage onto the cytosol can bind with cyclic GMP‐AMP synthase to catalyze the synthesis of cyclic GMP‐AMP and subsequently promote the activation of the stimulator of interferon gene (STING), a crucial factor contributing to renal inflammation, injury, and fibrosis. A recent study investigated the therapeutic effect of UA in a fructose‐induced hyperuricemic nephropathy mouse model. The results indicated that UA ameliorated hyperuricemic nephropathy by impairing the STING‐NLRP3 axis‐mediated inflammatory response via Parkin‐dependent mitophagy (Zhang et al. 2022).

### 1.5 Mito‐Protective Effect of EA in the Cardiovascular System

#### 1.5.1 Acrylamide Cardiotoxicity

Acrylamide, a harmful byproduct, is generated during food and industrial processes. Exposure to acrylamide can occur through inhalation, dermal contact, and ingestion, posing potential risks to human health (Salimi et al. 2021). Acrylamide is readily absorbed and distributed in the body. Glycidamide, the highly reactive metabolite of acrylamide, interacts with macromolecules like lipids, DNA, and proteins (Salimi et al. 2021). Oxidative stress and mitochondrial disruption have been proven to be the primary causes of acrylamide‐induced toxicities. Salimi et al. showed that acrylamide treatment disrupted mitochondrial function in human lymphocytes, indicated by an increase in ROS and MDA formation and a reduction in GSH and MMP (Salimi et al. 2021). They further showed that EA treatment reduced mitochondrial toxicity markers in human lymphocytes (Salimi et al. 2021).

#### 1.5.2 Bevacizumab Cardiotoxicity

Bevacizumab is an anti‐angiogenic drug used for the treatment of various cancers, specifically lung cancer. Besides its effectiveness for cancer treatment, recent evidence shows that bevacizumab treatment is associated with cardiovascular adverse effects. Mitochondrial damage has been proposed as the primary cause of bevacizumab‐induced cardiotoxicity (Li et al. 2021). A recent study on isolated rat heart mitochondria showed that bevacizumab reduced mitochondrial complex II activity. Moreover, mitochondrial swelling, MMP reduction, and elevated ROS formation were observed after bevacizumab treatment. EA treatment reversed all the above parameters (Khanlou et al. 2022).

#### 1.5.3 Clozapine Cardiotoxicity

Clozapine is an effective treatment for schizophrenia, but its negative impact on the cardiovascular system remains a significant concern (Layland et al. 2009). Complications such as myocarditis, cardiomyopathy, and pericarditis have been reported after clozapine treatment (Layland et al. 2009). A recent study on freshly isolated adult rat ventricular cardiomyocytes showed that EA treatment ameliorated the cytotoxic effects of clozapine by reducing ROS and MDA overproduction. It also improved lysosomal membrane integrity, MMP, and increased GSH content (Ahangari et al. 2022).

#### 1.5.4 Celecoxib Cardiotoxicity

Celecoxib, as an effective cyclooxygenase‐2 inhibitor, is used for the treatment of mild to moderate pain and inflammatory conditions (Eleiwa et al. 2024). It has been suggested that chronic use of celecoxib is associated with cardiovascular complications. A study showed that EA could reduce celecoxib cardiotoxicity through mitochondrial function enhancement. EA treatment increased MMP, GSH, and SDH activity while reducing ROS and lipid peroxidation in isolated mitochondria (Atashbar et al. 2021).

#### 1.5.5 Doxorubicin Cardiotoxicity

Dox, along with its hepatotoxicity, is also cardiotoxic. Several lines of evidence have shown that Dox cardiotoxic effects are associated with mitochondrial injuries. Bcl‐2 interacting protein 3 (BNIP3) is a mitochondrial protein essential for cell apoptosis. It has been shown that Bnip3 is essential for provoking oxidative injury in mitochondria following Dox treatment. Dynamin‐related protein 1 is another mitochondrial protein that plays a key role in mitochondrial fission. Dox‐treated cardiomyocytes showed excessive mitochondrial fragmentation (Dhingra et al. 2017). EA treatment reduced mitochondrial Bnip3 in cells treated with Dox. EA also reduced mitochondrial fission induced by Dox. In addition, another key finding of this study was that mitophagy induced by Dox was inhibited by EA treatment. In summary, the study revealed that EA prevented mitochondrial targeting of Bnip3 by Dox in cardiomyocytes. This is essential for subsequent events such as mitochondrial fission and mitophagy (Dhingra et al. 2017).

#### 1.5.6 Isoproterenol Cardiotoxicity

Isoproterenol (ISO) is a drug used for bradycardia. It has been shown that ISO can induce myocardial infarction in experimental models (Kannan and Quine 2012). A study using the heart tissue of rats showed that ISO exposure altered mitochondrial enzymes such as isocitrate dehydrogenase (ICDH), succinate dehydrogenase (SDH), malate dehydrogenase (MDH) and α‐ketoglutarate dehydrogenase (α‐KGDH). While ISO exposure showed deleterious effects on the Krebs cycle, EA pretreatment alleviated these effects. The structure of mitochondria was also improved by EA treatment. EA improved the structure of cristae with less vacuolar degeneration and swelling (Kannan and Quine 2012).

### 1.6 Mito‐Protective Effect of Urolithins on the Cardiovascular System

#### 1.6.1 Metabolic Cardiomyopathy

Metabolic cardiomyopathies develop in a wide spectrum of pathological conditions. They include a number of inherited metabolic diseases in early childhood affecting the heart and other organs. Cardiomyopathy arises from impaired energy production caused by defects in the metabolism of glycogen, lipids, and mucopolysaccharides, resulting in increased oxidative stress and mitochondrial dysfunction (Guertl et al. 2000; Huang et al. 2023). It was indicated that the expression of genes involved in mitophagy, such as *Pink1, Park2, MFN1, Atg9*, and *Rab7*, was downregulated in animal models of metabolic cardiomyopathy. UA treatment alleviated mitochondrial defects by improving mitochondrial respiratory capacity, MMP collapse, mitochondrial structure, and upregulation of mitophagy‐related genes (Guertl et al. 2000).

#### 1.6.2 Diabetic Cardiomyopathy

Diabetic cardiomyopathy is characterized by left ventricular stiffness, which results in impaired heart function due to the overproduction of ROS and cardiac remodeling activation (Li et al. 2024). New research has highlighted the importance of mitochondrial dysfunction and excessive ROS production in diabetic cardiomyopathy (Li et al. 2024). In this sense, Albasher et al. demonstrated that UA treatment exerts cardioprotective effects through Keap1 downregulation and Nrf2/HO‐1 activation. UA treatment also reduced apoptosis through Bax downregulation. Moreover, UA treatment activated SIRT1, which deacetylated forkhead box transcription factor1 (FOXO1), Nrf2, NF‐ĸB, and p53 (Albasher et al. 2022).

### 1.7 Mito‐Protective Effect of EA on the Nervous System

#### 1.7.1 Arsenic Neurotoxicity

Arsenic easily penetrates the blood–brain barrier, accumulating in the brain and causing neurotoxic effects (Abdollahzade et al. 2021). Arsenic exposure impairs brain function. The disruption of short‐term memory and learning after arsenic exposure has been reported in several studies (Vahidnia et al. 2007). Firdaus et al. revealed that EA treatment ameliorated arsenic neurotoxicity in the rat's hippocampus. EA exposure reduced ROS overproduction induced by arsenic dose‐dependently. Moreover, EA reversed arsenic‐mediated MMP disruption. Since mitochondrial membrane depolarization is considered the first trigger of apoptosis, it is assumed that arsenic exposure initiated apoptosis. In this regard, EA exposure exhibited significant anti‐apoptotic effects by downregulation and upregulation of Bax and Bcl‐2, respectively (Firdaus et al. 2018a). Furthermore, EA reduced the mRNA levels of pro‐inflammatory markers such as TNF‐α, IFNγ, and IL‐1β (Firdaus et al. 2018a). In line with this study, Firdaus et al. evaluated the neuroprotective effects of EA on SH‐SY5Y cells. They showed that EA treatment increased cell viability and reduced ROS generation, mitochondrial membrane depolarization, and cytochrome c release in SH‐SY5Y cells (Firdaus et al. 2018b).

#### 1.7.2 Cuprizone Neurotoxicity

Cuprizone is usually used to produce toxic demyelination that resembles the demyelination that occurs in multiple sclerosis (MS). A study showed that EA treatment improved a number of mitochondrial parameters, which play a key role in MS pathology. EA treatment improved muscle mitochondrial function. It also reduced mitochondrial ROS levels and upregulated the protein expression of Sirt3, which is a mitochondrial sirtuin that controls mitochondrial function. Additionally, EA effects on mitochondrial complexes and ATP levels were evaluated. EA treatment increased the activity of complex II, III, IV, and ATP (Khodaei et al. 2019).

#### 1.7.3 Rotenone Neurotoxicity

Rotenone, a widely used pesticide, has been shown to cause Parkinson's‐like effects in experimental models. A recent study in *Drosophila melanogaster* showed that rotenone exposure increased oxidant levels such as hydrogen peroxide, nitric oxide, and lipid peroxide (Adedara et al. 2023). Moreover, rotenone disrupted mitochondrial complex I activity and reduced the bioenergetic state. EA treatment ameliorated rotenone‐induced mitochondrial toxicity and induced significant antioxidant effects (Adedara et al. 2023).

### 1.8 Mito‐Protective Effect of Urolithins on the Nervous System

#### 1.8.1 Alzheimer's Disease

AD has been associated with mitochondrial dysfunction. However, the precise molecular mechanism(s) underlying impaired mitochondrial homeostasis in AD remain underinvestigated (Zhang et al. 2024). Recent studies have indicated the potential role of natural compounds in the prevention and treatment of AD (H. Li et al. 2022). Moreover, given that mitophagy deficits are prominent in mitochondrial dysfunction associated with AD, promoting mitochondrial integrity through mitophagy induction may serve as an effective intervention for AD (Jayatunga et al. 2021). In this respect, Hou et al. reported that long‐term UA treatment induced mitophagy through increasing lysosomal functions by regulation of cathepsin Z protein in AD transgenic mice (Hou et al. 2024). Another study in an in vivo AD model showed that UA treatment enhanced the expression of genes necessary for mitochondrial biogenesis, including transcription factor A and estrogen‐related receptors (Esselun et al. 2021).

#### 1.8.2 Parkinson's Disease

Mitochondrial dysfunction plays a vital role in the development of neurodegenerative disorders such as PD. There is substantial potential in treatment approaches that focus on addressing mitochondrial dysfunction for managing PD (Liu et al. 2022a). In a recent study, UA treatment reduced 6‐hydroxydopamine‐induced mitochondrial dysfunction in PC12 cells by enhancing mitochondrial biogenesis through upregulation of the SIRT1/PGC‐1α signaling pathway (Liu et al. 2022a).

#### 1.8.3 Ischemic Stroke

Ischemic stroke, characterized by a blockage in the blood vessels supplying the brain, is a leading cause of both mortality and long‐term disability globally (Chen et al. 2025). The exact pathological processes causing ischemic damage to neurons are still unclear (Feske 2021). Preventing cell apoptosis and reducing brain injury caused by ischemia/reperfusion is crucial. UA treatment showed anti‐apoptotic effects through downregulation of Bax and caspase‐3 with simultaneous upregulation of Bcl‐2 after cerebral ischemia (Lin et al. 2020).

## 2 Discussion

**FIGURE 2:**
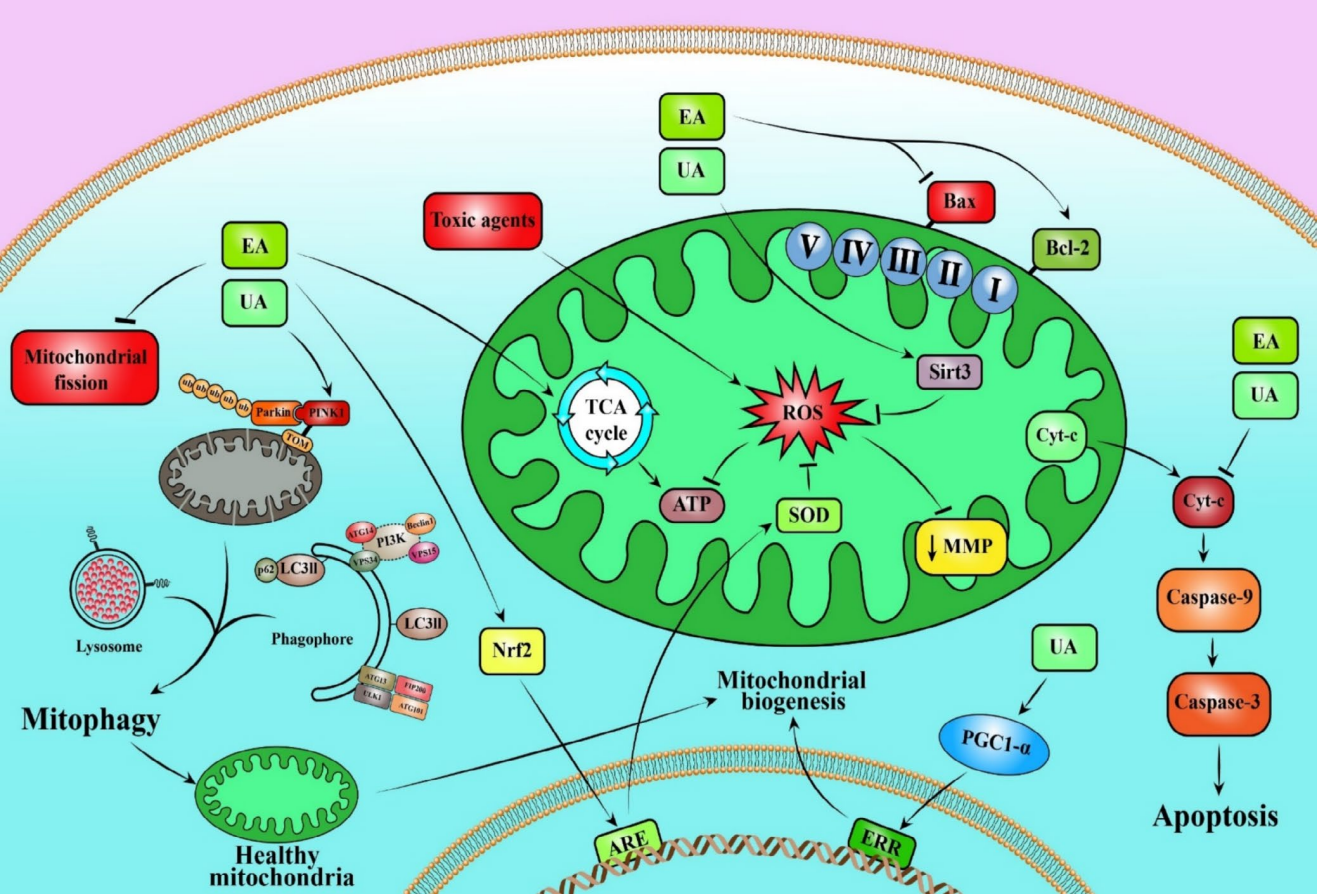
A schematic illustration of mechanism(s) insight of the ellagic acid and urolithin A mitochondrial‐protective effects. ↑ and → present the promote/activate, ⊥ and ↓ present the inhibitory/suppressive effects. Bax, Bcl‐2‐associated X protein; Cytochrome c, Cyt‐c; EA, Ellagic acid; ERR, Estrogen‐related receptor; MMP, Mitochondrial membrane potential; Nrf2, Nuclear factor erythroid 2‐related factor 2; PGC‐1α, Peroxisome proliferator‐activated receptor gamma; UA, Urolithin A.

**FIGURE 3:**
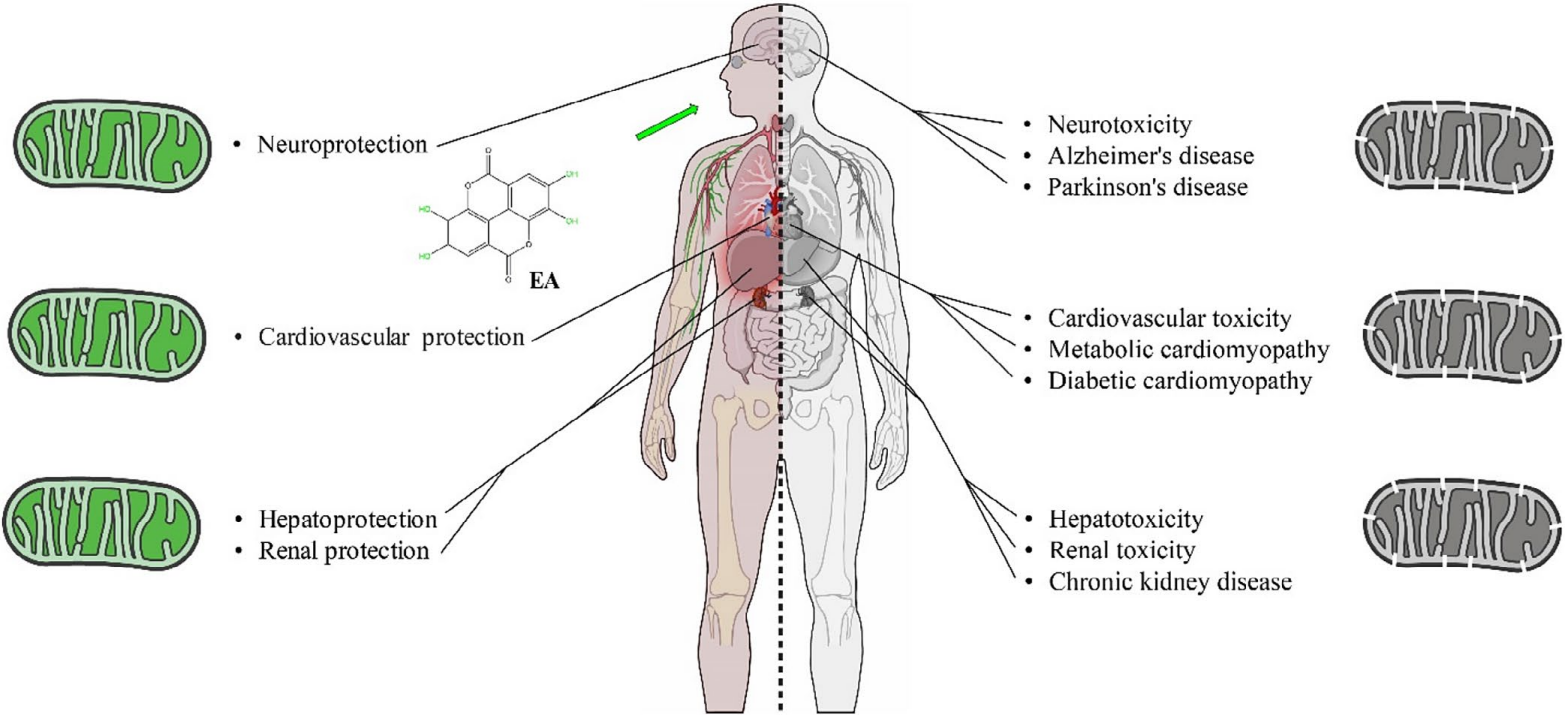
A schematic illustration of EA protection in various organ injuries.

For a long time, polyphenolic phytochemicals were believed to protect cells from oxidative damage by scavenging free radicals (Li et al. 2025; Sariözkan et al. 2016; Türk et al. 2021). For centuries, people around the world have pursued solutions for disease treatment through the utilization of medicinal plants (Wang et al. 2024a). Among these natural compounds, EA and its gut microbiota metabolites have garnered significant attention due to their diverse biological properties (Rahmani et al. 2023a, 2023b). Evidence from epidemiological studies suggests that foods rich in EA may protect against various human diseases. According to our literature review, EA, due to its specific chemical structure, having four hydroxyl groups and two lactone groups, exhibits antioxidant properties as indicated in several studies (Kannan and Quine 2012). In the human body, mitochondria produce energy and generate ROS. They produce superoxide anions as electrons leak from complexes I and III of the mitochondrial respiratory chain. The superoxide anion and its derivative, hydrogen peroxide, fall under the category of mitochondrial reactive oxygen species (mtROS). Under normal physiological conditions, mtROS are efficiently neutralized by a cellular antioxidant defense system, which includes SOD, catalase (CAT), and glutathione peroxidase (GPx). However, under pathological conditions, mtROS overproduction occurs, leading to oxidant radical accumulation. This can harm mitochondria and cells, affecting cellular health. It seems that EA not only acts on multiple biological defenses through the direct and indirect effects of its significant antioxidant properties, but it also contributes to the maintenance and enhancement of mitochondrial activity by directly acting on mitochondrial enzymes and complexes. Several studies showed that some toxicants, such as arsenic (Keshtzar et al. 2016), acrylamide (Salimi et al. 2021), and rotenone (Adedara et al. 2023), can harm mitochondria in various tissues. However, EA treatment promoted beneficial effects on cellular antioxidant defense (Figures 2 and 3) (Adedara et al. 2023; Sepand et al. 2016). Even though the exact sequence of events linking oxidative stress and mitochondrial dysfunction has yet to be fully understood, it is suggested that heightened oxidative stress leads to mitochondrial dysfunction, which in turn raises the levels of mtROS. When the level of ROS inside the cell rises, the disturbance of mitochondria leads to a positive feedback loop, causing increased ROS production, a phenomenon known as ROS‐induced ROS release. This concept, which involves an ongoing cycle of ROS generation, is currently acknowledged as a component of the mechanism underlying ROS‐related diseases. Morphological changes and functional losses of the mitochondria represent mitochondrial dysfunction caused by oxidative damage. The swelling and fragmentation of mitochondria are commonly observed as structural changes. The disruption of the MMP indicates mitochondrial dysfunction. When the MMP is lost, it causes problems in the mitochondrial electron transport chain, reduces metabolic oxygen consumption, leads to ATP depletion, and lowers energy metabolism. Oxidative stress on the mitochondria can lead to permeabilization, which triggers the apoptotic pathway. Specifically, the opening of the mPTP causes cytochrome c to be released into the cytoplasm, activating pro‐apoptotic caspases (Figures 2 and 3). In our literature review, EA treatment showed positive effects on mitochondrial function and structure. Moreover, several studies showed that EA was able to exert anti‐apoptotic effects through the regulation of mitochondrial apoptosis‐related pathways (Figures 2 and 3) (Ebrahimi et al. 2019; Karim et al. 2023; Sepand et al. 2016). Urolithins are metabolites derived from the microbial transformation of EA and ellagitannins in the gut. Among the urolithins, UA has garnered significant attention in recent years leading to numerous studies on this compound. The safety assessment indicates that the substance is safe in both human trials at doses ranging from 250 to 200 mg/kg and in animal studies, specifically in mice, at doses ranging from 1 to 450 mg/kg (Hou et al. 2024). We found evidence suggesting that urolithins may have mito‐protective effects based on various in vivo and in vitro studies. It has been observed that UA not only exhibits antioxidant effects through the regulation of mitochondrial antioxidant enzymes but also enhances mitochondrial dynamics, particularly the process of mitophagy, which plays a crucial role in maintaining mitochondrial health (Rahmani et al. 2024a). Mitophagy is the process of selectively removing dysfunctional mitochondria through autophagy (Deng et al. 2024). The procedure enhances the quality of the cellular mitochondria and is connected to generating new organelles (Deng et al. 2024). The decline of the procedure is linked to aging and various age‐related illnesses (Rahmani et al. 2024a). The hindrance of mitophagy affects mitochondrial balance and may lead to the accumulation of impaired organelles, causing harm to the cells (Rahmani et al. 2024a). In line with this, studies showed that UA treatment enhanced PINK1/Parkin‐dependent mitophagy, leading to improvement of mitochondrial defects in neurodegenerative and cardiomyopathy models (Figures 2 and 3) (Hou et al. 2024; Huang et al. 2023). Although recent studies have shown the positive impact of UA on human mitochondrial function, additional confirmatory studies are necessary to validate these findings (Liu et al. 2022b; Singh et al. 2022).

## 3 Conclusion and Perspective

Dietary antioxidants like urolithins and their precursor EA have shown protective effects in experimental models of diseases, particularly due to their antioxidant properties and mito‐protective effects. These compounds present diverse biological functions, making them promising candidates for developing novel pharmacological treatments. Both EA and urolithins have shown broad pharmacological applications in various in vivo and in vitro studies. These findings suggest that EA and urolithins confer beneficial effects in various disease models through their mito‐protective effects, which could potentially be harnessed in the treatment of mitochondrial‐related diseases in humans. However, further research is required to validate their efficacy in human studies.

## Author Contributions

**Sohrab Rahmani:** writing – original draft (equal). **Ali Roohbakhsh:** writing – review and editing (equal). **Yazdan Hasani Nourian:** writing – original draft (equal), writing – review and editing (equal). **Gholamreza Karimi:** conceptualization (equal), writing – review and editing (equal).

## Ethics Statement

The authors have nothing to report.

## Consent

The authors have nothing to report.

## Conflicts of Interest

The authors declare no conflicts of interest.

## Data Availability

Data are available as request.
